# Coenzyme Q_10_ dose-escalation study in hemodialysis patients: safety, tolerability, and effect on oxidative stress

**DOI:** 10.1186/s12882-015-0178-2

**Published:** 2015-11-03

**Authors:** Catherine K. Yeung, Frederic T. Billings, Adam J. Claessens, Baback Roshanravan, Lori Linke, Mary B. Sundell, Suhail Ahmad, Baohai Shao, Danny D. Shen, T. Alp Ikizler, Jonathan Himmelfarb

**Affiliations:** Department of Pharmacy, University of Washington School of Pharmacy, Box 357630, Seattle, WA 98195 USA; Department of Medicine, Kidney Research Institute, University of Washington School of Medicine, Box 359606, Seattle, WA 98195 USA; Division of Critical Care Medicine, Department of Anesthesiology, Vanderbilt University Medical Center, 1211 21st Avenue South, 526 Medical Arts Building, Nashville, TN 37205 USA; Diabetes and Obesity Center of Excellence, Department of Medicine, University of Washington, Box 358055, Seattle, WA USA; Division of Nephrology, Department of Medicine, Vanderbilt University Medical Center, 1161 21st Avenue South, S-3223 Medical Center North, Nashville, TN 37232 USA

**Keywords:** Clinical study, Coenzyme Q_10_, Hemodialysis, Kidney disease, Oxidative stress

## Abstract

**Background:**

Coenzyme Q_10_ (CoQ_10_) supplementation improves mitochondrial coupling of respiration to oxidative phosphorylation, decreases superoxide production in endothelial cells, and may improve functional cardiac capacity in patients with congestive heart failure. There are no studies evaluating the safety, tolerability and efficacy of varying doses of CoQ_10_ in chronic hemodialysis patients, a population subject to increased oxidative stress.

**Methods:**

We performed a dose escalation study to test the hypothesis that CoQ_10_ therapy is safe, well-tolerated, and improves biomarkers of oxidative stress in patients receiving hemodialysis therapy. Plasma concentrations of F_2_-isoprostanes and isofurans were measured to assess systemic oxidative stress and plasma CoQ_10_ concentrations were measured to determine dose, concentration and response relationships.

**Results:**

Fifteen of the 20 subjects completed the entire dose escalation sequence. Mean CoQ_10_ levels increased in a linear fashion from 704 ± 286 ng/mL at baseline to 4033 ± 1637 ng/mL, and plasma isofuran concentrations decreased from 141 ± 67.5 pg/mL at baseline to 72.2 ± 37.5 pg/mL at the completion of the study (*P* = 0.003 vs. baseline and *P* < 0.001 for the effect of dose escalation on isofurans). Plasma F_2_-isoprostane concentrations did not change during the study.

**Conclusions:**

CoQ_10_ supplementation at doses as high as 1800 mg per day was safe in all subjects and well-tolerated in most. Short-term daily CoQ_10_ supplementation decreased plasma isofuran concentrations in a dose dependent manner. CoQ_10_ supplementation may improve mitochondrial function and decrease oxidative stress in patients receiving hemodialysis.

**Trial Registration:**

This clinical trial was registered on clinicaltrials.gov [NCT00908297] on May 21, 2009.

## Background

Five hundred thousand patients in the United States receive maintenance hemodialysis (MHD) for end-stage renal disease (ESRD) [1]. Life expectancies for MHD patients are 17–34 % less than those of the general population [2]. Some of this excess mortality may be attributable to an increased risk of cardiovascular disease as a result of increased oxidative stress [3, 4]. Oxidative stress can originate from multiple sources, including the decoupling of the electron transport chain in the mitochondria and inflammation-mediated production of superoxide via NADPH-oxidase and resulting altered oxygen handling capacity.

Coenzyme Q_10_ (CoQ_10_) is a required component of the mitochondrial electron transport chain, where it transfers electrons from complexes 1 and 2 to complex 3. Reduction and oxidation of CoQ_10_ also reduces lipid radicals and oxidizes superoxide. CoQ_10_ depletion leads to inefficient electron transport, increased reactive oxygen species (ROS) production, decreased adenosine triphosphate (ATP) production, and altered mitochondrial membrane potential [5]. CoQ_10_ treatment, on the other hand, improves mitochondrial coupling of respiration to oxidative phosphorylation, decreases superoxide production in endothelial cells, and may improve functional cardiac capacity in patients with congestive heart failure [6]. In addition to the effects on mitochondrial transport, CoQ_10_ also exerts global antioxidant effects, with the reduced form able to react directly with free radicals, wherein it is converted to the oxidized form [7]. For these reasons, CoQ_10_ is frequently administered as a dietary supplement in alternative and complementary therapy, but no studies have examined high dose CoQ_10_ tolerability and efficacy in dialysis patients, a population with increased oxidative stress [8, 9]. F_2_-isoprostane and isofuran concentrations, products of non-enzymatic arachidonic acid peroxidation, are considered one of the most reliable approaches for assessing systemic oxidative stress in vivo [10]. The formation of F_2_-isoprostanes and isofurans is differentially regulated by oxygen tension; the formation of F_2_-isoprostanes is favored at low oxygen tensions whereas the formation of isofurans is favored at high oxygen tensions, as occurs in the setting of mitochondrial dysfunction [11, 12]. Plasma F_2_-isoprostane concentrations have been shown to be two to four times higher in dialysis patients than in age- and gender- matched healthy subjects [13, 14], consistent with the increased oxidative burden in this population. Plasma isofuran concentrations have not previously been reported in dialysis patients.

The present study tested the hypothesis that oral CoQ_10_ administration is safe, well-tolerated, and decreases oxidative stress in MHD patients, with the additional goals of determining the maximum well-tolerated dose of CoQ_10_.

## Methods

### Study design

**Fig. 1:**
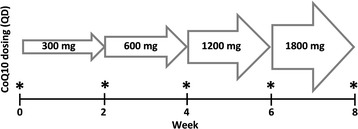
CoQ_10_ dose escalation study design. *Measurement of plasma CoQ10, comprehensive metabolic panel, creatine phosphokinase, and oxidative stress biomarkers

We performed an open label, dose-escalation study (Fig. 1) to evaluate the safety and tolerability of CoQ_10_ in HD patients using a commercially available CoQ_10_ chewable wafer (Vitaline Formulas, Wilsonville, OR).

### Study population

End stage renal disease patients, between the ages of 18 and 85 years, receiving thrice weekly dialysis using high-flux dialyzers, with a life expectancy of >1 year, and a baseline plasma F_2_-isoprostane concentration greater than or equal to 50 pg/ml were eligible for study. Twenty MHD subjects were enrolled from two university medical centers. All subjects provided written informed consent that was approved by the University of Washington and the Vanderbilt University Institutional Review Boards. All study procedures were conducted in accordance with Helsinki Declaration of 1975 (as revised in 2000).

Subject exclusion criteria included: history of poor adherence to MHD or medical regimen, AIDS (HIV seropositivity was not an exclusion criteria), active malignancy excluding basal cell carcinoma of the skin, gastrointestinal dysfunction requiring parenteral nutrition, kidney transplant < 6 months prior to study entry or anticipated live donor kidney transplant, current use of vitamin E supplements > 60 IU/day, vitamin C supplements > 150 mg/day, current use of l-carnitine or other antioxidant or nutritional supplements, initiation of hemodialysis within 90 days prior to study enrollment, hospitalization within the past 60 days, dialysis with a tunneled catheter as a temporary vascular access, and a history of a major atherosclerotic event (defined as myocardial infarction, urgent target-vessel revascularization, coronary artery bypass surgery, or stroke) within six months.

Plasma samples from healthy control subjects (*n* = 10) were randomly selected from the Kidney Research Institute biorepository. Healthy control subjects had normal kidney function and were not taking statin medications. Healthy control subjects were not matched for age, race, or gender with subjects receiving CoQ_10_ supplementation. The control subjects were younger, more likely to be female, and less likely to be taking a statin than the dialysis subjects.

With a sample size of 20 subjects, we predicted that we would have at least 94 % power to detect a 45 % change in plasma F_2_-isoprostane levels after CoQ_10_ administration (estimated mean plasma F_2_-isoprostane concentrations in MHD patients = 96 pg/mL, with a standard deviation of 49 pg/mL), assuming a 2-sided, 0.05 alpha level using a paired *t*-test approach.

### CoQ_10_ dose escalation

Subjects were administered 300 mg CoQ_10_ for 14 days and then 600, 1200, and 1800 mg CoQ_10_ daily, each for 14 days. Subjects returned to the clinic at the end of each dosing period and spontaneously reported adverse events were recorded at every visit. Blood samples were collected at baseline, and after each 14-day course of treatment for determination of comprehensive metabolic panel (electrolytes, blood urea nitrogen, creatinine, glucose, albumin, total protein, calcium, alkaline phosphatase, ALT, AST, total bilirubin), creatine phosphokinase (CPK), F_2_-isoprostane and isofuran concentrations. Blood samples for High Density Lipoprotein (HDL) apoA-1 Met(O) were collected at baseline and following 1200 mg dosing period. All blood samples were immediately chilled, and then centrifuged within one hour of collection at 2500 RPM, 4 °C, for 15 min. Plasma (heparinized) was removed and stored at −80 °C until analysis. Ten subjects participated in an additional study in which CoQ_10_ levels were measured prior to and immediately following standard HD to assess the effect of HD on plasma CoQ_10_ concentrations. CoQ_10_ concentrations were also measured in the plasma of the 10 unmatched healthy control subjects. Access to the remaining samples may be granted by contacting the corresponding author.

### Assays

For CoQ_10_ measurement, plasma samples were thawed on ice, and a 100 μl aliquot was mixed with 200 μL ice cold 1-propanol containing Coenzyme Q_9_ (CoQ_9_) and CoQ_9_H_2_ as internal standards. Precipitated proteins were removed by cold centrifugation, and the supernatant injected directly onto the LC-MS/MS for analysis. Samples in the autosampler were held at 4 °C until injection. Chromatography of the analytes was accomplished using an Agilent 1200s-series LC system equipped with a Zorbax SB-C_18_ column (30 mm × 2.1 mm × 3.5 μM particle size) that was maintained at 40 °C. The mobile phase consisted of a binary gradient of methanol and 5 mM ammonium formate pumped at a flow rate of 0.8 mL/min. The MS/MS was operated in positive ESI mode with nitrogen as drying gas at a flow of 10 L/min and 350 °C, and with nitrogen nebulizer gas set at 35 psi. The monitored transitions for CoQ_10_H_2_, CoQ_10_, CoQ_9_H_2_ and CoQ_9_ were *882.7 → 197.1, 863.7 → 197.1, 814.7 → 197.1,* and *795.6 → 197.1* respectively. Method validation, including stability of analytes and internal standards, and comparisons to established methods are detailed in reference [15].

For F_2_-isoprostane and isofuran measurements, internal standard [^2^H_4_]-15-F_2T_-isoprostane was added to each plasma sample prior to C-18 and silica solid phase extraction, thin layer chromatography, and derivatization to penta-fluorobenzyl ester, trimethylsilyl ether derivative [16]. GC/NICI-MS was performed (Agilent 5973) using 15 m x 0.25 μm thick fused silica capillary columns (J and W Scientific). The major ion generated was m/z 569 carboxylate anion [M-181 (M-CH_2_C_6_F_5_)]. The corresponding ion generated from the [^2^H_4_]-15-F_2T_-isoprostane internal standard was m/z 573, and the isofuran ion was *m/z* 585.

For Met(O) measurement, HDL was isolated, digested and oxidation of Met residues in apoA-I of HDL was quantified by isotope dilution mass spectrometry (MS) and selective reaction monitoring (SRM) on a Thermo TSQ Vantage coupled to a Waters nanoACQUITY UltraPerformance liquid chromatography system as previously described [17].

Comprehensive metabolic panel and CPK measurements were performed in the CLIA-certified hospital laboratories of University of Washington Medical Center and Vanderbilt University Medical Center. Plasma IL-6 was measured using a commercial kit according to the manufacturer’s protocol (R&D Systems, Minneapolis MN).

### Data analysis

Mean baseline values and change from baseline were determined for CoQ_10_ concentrations, all safety endpoints (comprehensive metabolic panel, CPK, electrocardiogram results, physical examination findings), and efficacy measures (oxidative stress biomarker concentrations). Each of these parameters was analyzed via one-way repeated measures analysis of variance to determine the presence or absence of dose-related differences. In addition, mixed effects modeling was used to test the effect of increased CoQ_10_ dose and duration with fixed effect of time (dose), random subject effect, and an unstructured repeated covariance type. Study subject concentrations of CoQ_10_, F_2_-isoprostanes, and isofurans were also compared to concentrations in non-dialysis historical control subjects. The primary analysis included all subjects. Subgroup analyses were conducted in statin treated (*n* = 6) and non-statin treated (*n* = 14) subjects. Spearman’s correlation coefficients were used to assess correlations between plasma CoQ_10_ concentrations and oxidative stress biomarkers. Two-tailed *P*-values ≤ 0.05 were considered significant in all analyses. When appropriate, corrections were made for multiple comparisons. Statistical analysis was conducted using STATA 11.0 and SAS 9.1 (SAS Institute, Cary, NC, USA). Data is expressed as mean ± standard deviation unless otherwise specified.

## Results

**Fig. 2:**
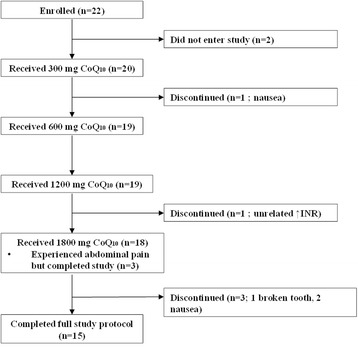
Overview of study subject participation and study withdrawal

Twenty subjects were recruited for the study, fifteen of whom completed the entire dose escalation protocol (Fig. 2). The mean age of subjects was 58 years with 8 females studied. Average length of time on dialysis was 8.0 ± 8.9 years (range 0.7 to 29.4 years). Seven subjects were taking statin medications. Three subjects discontinued study participation due to abdominal pain or nausea; one during the 300 mg/day period, one during the 1200 mg/day period, and one after the 1800 mg/day period. One subject discontinued participation after an unrelated elevation in INR (international normalized ratio), and one subject discontinued participation after damaging a tooth from chewing the CoQ_10_ wafer. Three subjects reported stomach discomfort at the 1800 mg/day dose but completed the study. One subject was found expired at home 22 days after completing the study. This death was attributed to natural causes and was deemed to be unrelated to study participation.

### Plasma CoQ_10_ concentrations

**Fig. 3:**
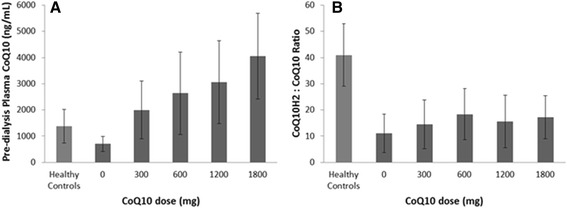
Effect of Coenzyme Q_10_ supplementation on plasma concentrations of total CoQ_10_ (**a**) and CoQ_10_(H_2_):CoQ10 ratios (**b**) (mean ± SD) in study subjects and unmatched healthy controls. Sample numbers for baseline (0 mg), 300 mg, 600 mg, 1200 mg, 1800 mg, and healthy controls were 20, 19, 19, 18, 15, and 10, respectively

**Fig. 4:**
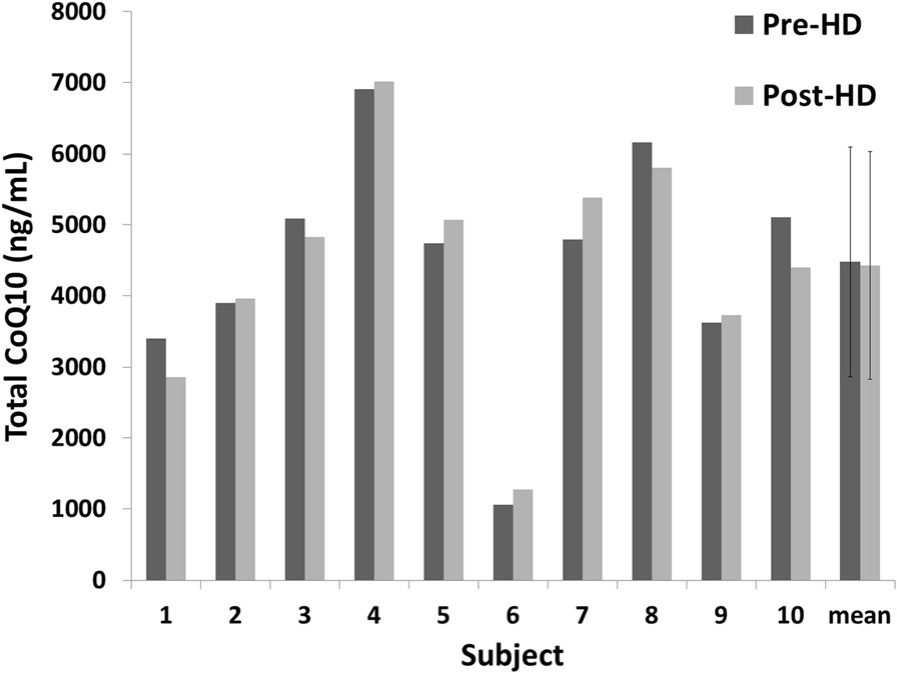
Effect of hemodialysis (HD) on plasma concentrations of CoQ_10_ in study subjects administered 1800 mg of CoQ_10_ for 14 days (*n* = 10). Dark bars indicate total CoQ_10_ levels prior to standard hemodialysis treatment, lighter bars are following hemodialysis. Error bars indicate SD of the means (paired *T*-test for means *p* = 0.72)

Mean plasma total CoQ_10_ levels at baseline (prior to a mid-week dialysis session) were 704 ± 286 ng/mL, significantly lower than CoQ_10_ levels in healthy control patients (1385 ± 640 ng/mL, *P* = 0.0014). Plasma CoQ_10_ concentrations increased linearly with escalation of CoQ_10_ dose (Fig. 3). At the end of the 1800 mg/day dosing period, total CoQ_10_ plasma concentrations averaged 4032 ± 1637 ng/mL (*P* < 0.001 compared to baseline and to healthy controls). The CoQ_10_ redox ratio (CoQ_10_H_2_:CoQ_10_) increased from 14 ± 7.4 at baseline to 22 ± 9 after supplementation (*P* = 0.013). Despite this increase, the redox ratio remained significantly less than that observed in healthy controls (41 ± 12, *P* < 0.001, Fig. 3). Statin using (*n* = 6) and non-using (*n* = 14) subjects had similar plasma concentrations of CoQ_10_ and CoQ_10_H_2_: CoQ_10_ ratios at baseline and at the end of the study (3438 ± 1899 ng/mL, redox ratio 25.7 ± 9.4 in statin users vs. 4080 ± 1467 ng/ml, redox ratio 18.8 ± 6.5 in statin non-users at the end of the study; *P* = 0.31 for CoQ_10_ plasma concentration, *P* = 0.10 for redox ratio). Plasma CoQ_10_ levels measured just before and immediately after hemodialysis did not differ significantly (Fig. 4), indicating that hemodialysis does not clear the lipophilic CoQ_10_ from blood or acutely alter the circulating pool of CoQ_10_ (*p* = 0.72).

### Effects of CoQ_10_ supplementation on F_2_-isoprostanes and Isofurans

**Fig. 5:**
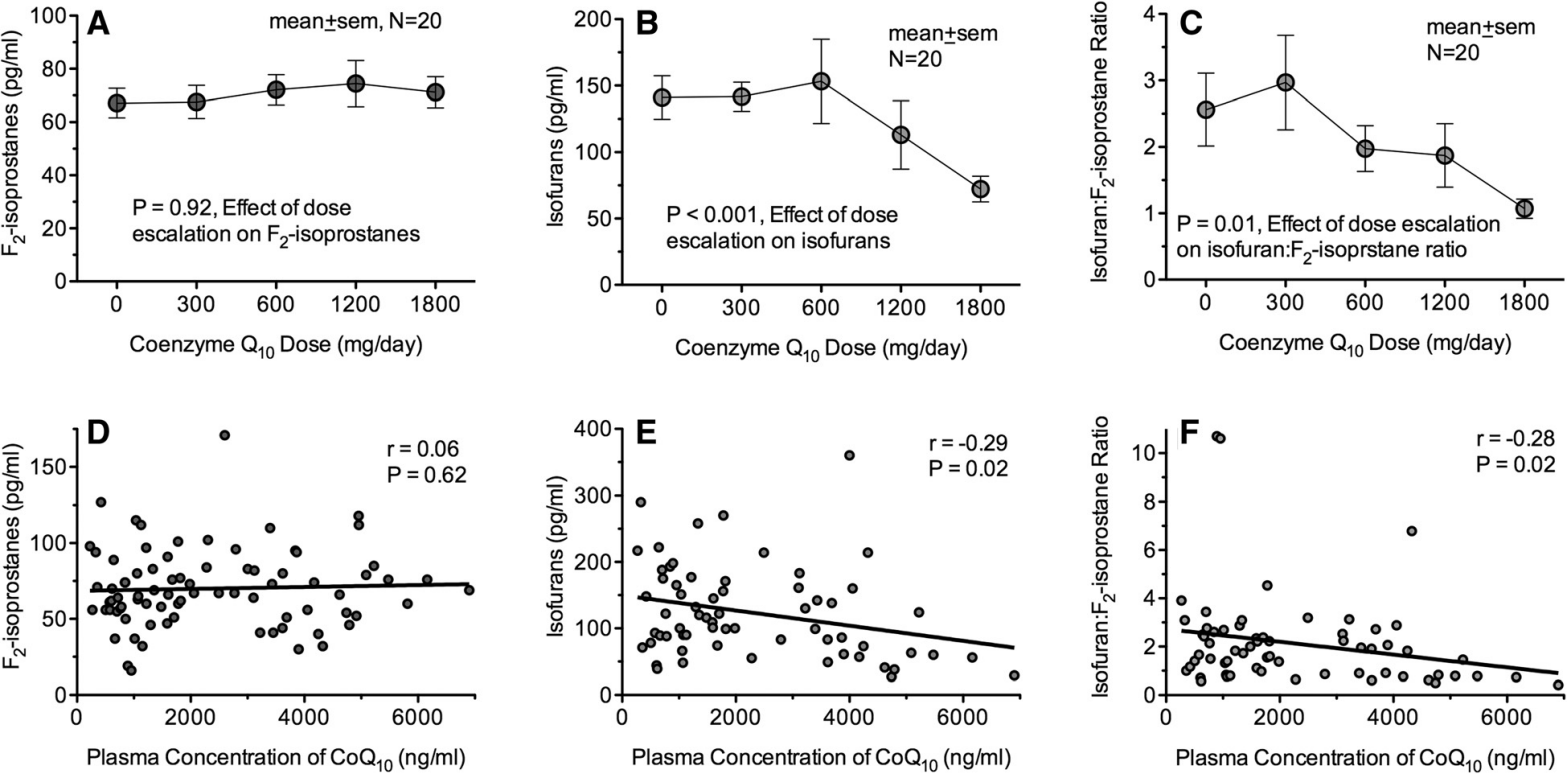
Effect of CoQ_10_ dose escalation (**a**–**c**) and total CoQ_10_ plasma concentrations (**d**–**f**) on plasma concentrations of F_2_-isoprostanes, isofurans, and isofuran:F_2_-isoprostane ratios

Baseline F_2_-isoprostane and isofuran levels were 66.9 ± 24.9 pg/ml and 141 ± 67.5 pg/ml respectively, notably higher than levels observed in 140 historic control patients not on dialysis (42.5 ± 32.6 pg/mL F_2_-isoprostanes, *P* = 0.002 and 57.0 ± 52.9 pg/mL isofurans, *P* < 0.001) [18]. During CoQ_10_ dose escalation, F_2_-isoprostane concentrations did not change compared to baseline (*P* = 0.92). Plasma isofuran concentrations, however, decreased following the 1200 mg (112.8 ± 72.9 pg/ml) and 1800 mg (72.8 ± 37.5 pg/ml) dosing periods (*P* < 0.001, effect of dose escalation on isofurans, Fig. 5a-c). Isofuran concentrations following 1800 mg/day CoQ_10_ dosing were 45.1 ± 8.1 % lower than baseline concentrations (*P* = 0.003). In addition, the ratio of isofuran concentrations to F_2_-isoprostane concentrations decreased from 2.56 ± 2.26 to 1.07 ± 0.56 during CoQ_10_ dose escalation (*P* = 0.01), a ratio similar to that observed in non-dialysis control subjects (1.53 ± 1.31, *P* =0.16). Consistent with the effect of CoQ_10_ dose escalation on isofuran concentrations and isofuran:F_2_-isoprostane ratios, plasma concentrations of CoQ_10_ were inversely correlated with isofuran concentrations and isofuran:F_2_-isoprostane ratios (CoQ_10_ isofuran correlation coefficient = −0.29, *P* = 0.02; CoQ_10_ isofurans:F_2_-isoprostanes ratio correlation coefficient = −0.28, *P* = 0.02). There was no correlation between plasma concentrations of F_2_-isoprostanes and plasma CoQ_10_ concentrations (*P* = 0.62, Fig. 5d–f).

### Effects of CoQ_10_ supplementation on interleukin-6 (IL-6)

**Fig. 6:**
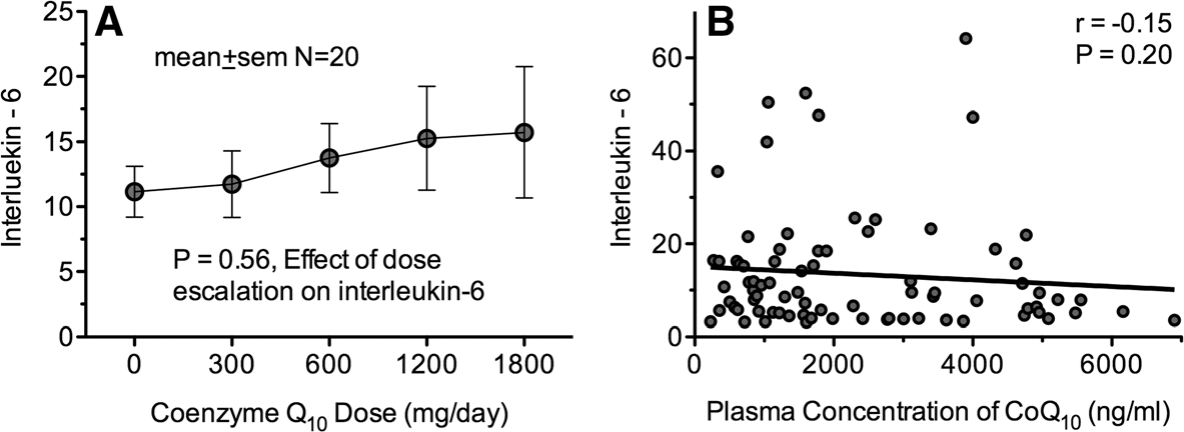
Effect of CoQ_10_ dose escalation (**a**) and total CoQ_10_ plasma concentrations (**b**) on plasma concentrations of interleukin-6

Baseline mean IL-6 concentration was 11.2 ± 8.1 ng/ml. During CoQ_10_ dose escalation, IL-6 levels did not change significantly (15.7 ± 18.9 ng/mL at 1800 mg CoQ_10_, *P* = 0.56), and no association was observed between IL-6 levels and plasma CoQ_10_ levels (*P* = 0.20) (Fig. 6a and b).

### Effects of CoQ_10_ supplementation on apoprotein A-1 (apoA-1) methionine oxidation (Met(O))

**Fig. 7:**
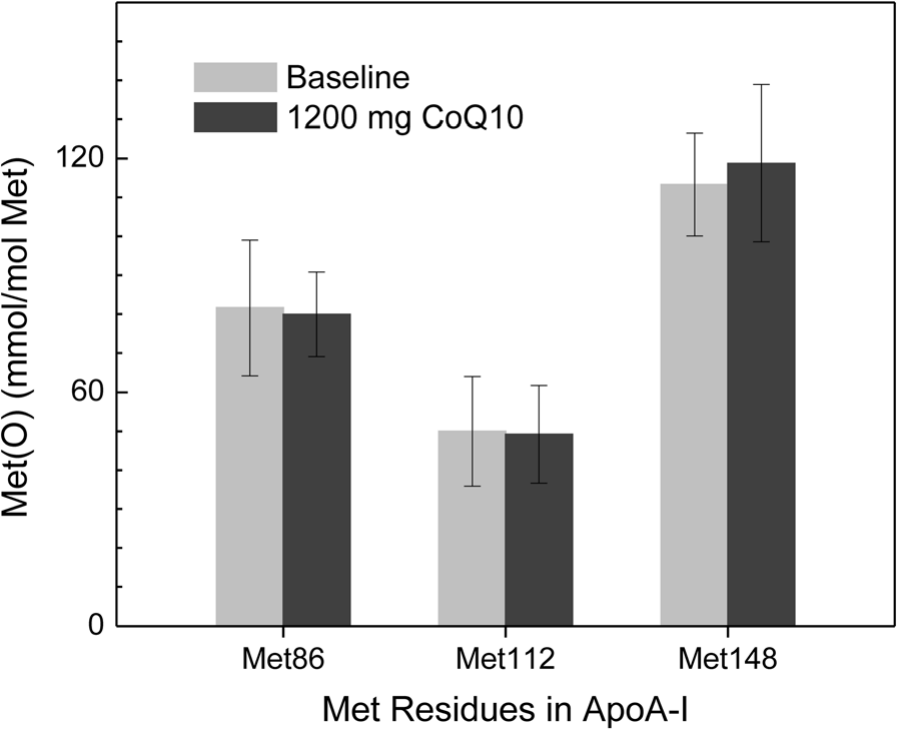
Effect of CoQ10 supplementation (following 1200 mg dosing period) on levels of oxidized methionine (mean ± SD) in apolipoprotein A-I of high density lipoprotein (all p-values > 0.5)

Oxidation of Met residues 86, 112, or148 in apoA-1 was not different following the 1200 mg CoQ_10_ dosing period, as compared to baseline apoA-1 Met oxidation (Fig. 7).

### Safety and tolerability

Six subjects complained of nausea and abdominal pain during the course of the study. One of these subjects made this complaint during the 300 mg dosing period, two following the 1200 mg dosing period, and three following the 1800 mg dosing period. Three of these subjects discontinued CoQ_10_ treatment, but the other three subjects continued taking CoQ_10_ despite reporting nausea and abdominal pain. Gastrointestinal complaints resolved after discontinuation of CoQ_10_. One subject (non-statin user) was found to have a CPK concentration of 2918 U/L following the 600 mg dosing period but was known to participate in competitive athletics and did not report any myalgia. He remained in the study and following the 1200 mg period his CPK level returned to 311 U/L (similar to his baseline concentration). Overall mean CPK concentrations did not change in the study cohort during CoQ_10_ dose escalation. CoQ_10_ dose escalation did not affect blood pressure.

## Discussion

In this dose escalation study we found oral administration of CoQ_10_ to be safe and well-tolerated at doses under 1800 mg/day. Three of the 5 of the adverse events occurred in subjects receiving more than 1200 mg daily, and only 3 of 6 subjects experiencing adverse effects withdrew from the study. Plasma CoQ_10_ levels increased linearly with dose, suggesting that the chewable wafer used in this study has consistent bioavailability. In addition, our data indicated that CoQ_10_ may reduce systemic oxidative stress in MHD patients in a dose-dependent manner.

CoQ_10_ has been studied extensively in healthy populations and patients with cognitive impairment. In these populations, CoQ_10_ has also been well tolerated. MHD patients are subject to elevated inflammation and oxidative stress as a result of ineffective clearance of uremic toxins, especially middle molecules (molecular weight 300–20,000 Da) [19] that include circulating cytokines. We have previously observed that MHD patients have 63 % higher concentrations of F_2_-isoprostanes than healthy controls [9], and a reduction in oxidative stress has been associated with better outcomes in MHD patients. A study by Sakata et al. evaluated the efficacy of a low oral dose of CoQ_10_ (100 mg daily) for 6 months in Japanese MHD patients and demonstrated that CoQ_10_ partially suppressed advanced oxidation protein product accumulation [20]. Singh et al. also demonstrated a benefit of CoQ_10_ in CKD; subjects who received 60 mg of CoQ_10_ three times daily had reduced serum creatinine and urea concentrations, increased creatinine clearance, and urine output compared with controls [21]. The doses used in Sakata and Singh studies were lower than those typically used in other studies (300–1200 mg daily); higher doses of CoQ_10_ may be required to substantively reduce oxidative stress in the MHD population. The present study is the first study to evaluate the safety and tolerability of high dose CoQ_10_ in a population receiving MHD.

CoQ_10_ dose escalation reduced plasma concentrations of isofurans but not F_2_-isoprostanes or HDL apoA-1 Met oxidation. To our knowledge, this is the first in vivo study in any patient population to demonstrate that plasma concentrations of isofurans are modifiable by any therapeutic antioxidant strategy. Our results are consistent with previously published unrelated studies, that show that plasma F_2_-isoprostane concentrations are significantly increased in patients undergoing maintenance HD compared with healthy subjects (published healthy controls: 37.6 pg/mL [14], subjects at baseline in this study: 65 pg/mL, after 1800 mg CoQ10: 72 pg/mL). In contrast, our subjects who also had elevated concentrations of isofurans at baseline (0.145 ng/mL) compared with healthy subjects (0.071 ng/mL) [22] demonstrated near-normalization after maximal supplementation (0.078 ng/mL). The generation of isofurans and F_2_-isoprostanes is regulated by the availability of oxygen; in cellular environments with relatively low oxygen availability, F_2_-isoprostanes are generated from ROS, while isofurans are preferentially generated in environments with relatively high oxygen availability. *In vivo*, isofuran generation is observed during inhalation of high concentrations of oxygen in healthy subjects and in tissues with low consumption of oxygen such as the substantia nigra of Parkinson’s patients [11]. Mitochondrial dysfunction observed in Parkinsonism or ESRD, may lead to high cellular oxygen tension since dysfunctional mitochondria consume little oxygen. In this environment ROS formation preferentially increases the generation of isofurans but not F_2_-isoprostanes. CoQ_10_ transfers electrons from complexes 1 and 2 to complex 3 in the mitochondrial electron transport chain. The suppression of isofuran generation observed with CoQ_10_ dose escalation is consistent with the idea that CoQ_10_ improves mitochondrial function in MHD patients and reduces the generation of ROS.

In order to differentiate the effects of CoQ_10_ on mitochondrial coupling versus direct antioxidant effect, we evaluated the effect of CoQ_10_ supplementation on ApoA-1 Met oxidation, which is selective for inflammation-mediated oxidative damage by myeloperoxidase (MPO). The lack of effect of CoQ_10_ escalation on apoA-1 Met oxidation in circulating HDL suggests the salutary effect of CoQ_10_ may be due to improved local mitochondrial function rather than a systemic anti-oxidant effect. The reduction of HDL-associated lipid hydroperoxides to the corresponding lipid hydroxides is responsible for specific oxidized Met residues (Met(O) 86 and Met(O) 112) in apoA-I, the major HDL protein [17]. In addition, hydrogen peroxide and hypochlorous acid (HOCl), a strong oxidant produced by the phagocyte heme enzyme myeloperoxidase may selectively oxidize Met112 and Met148 in apoA-I [23, 24]. In addition to the lack of effect of CoQ10 supplementation on ApoA-1 Met oxidation, no significant change in IL-6, a pro-inflammatory cytokine (11.2 ± 8.1 pg/mL at baseline; 15.7 ± 18.9 pg/mL at study conclusion (*p* = 0.56), in comparison to unrelated healthy controls with a median IL-6 concentration: 3.8 pg/mL) [25], was observed during this study, providing more presumptive evidence that the actions of CoQ_10_ are not due to a global anti- inflammatory effect. Taken together, our observations support the hypothesis that CoQ_10_ supplementation reduces mitochondrial oxidative stress, rather than by increasing general antioxidant or anti-inflammatory capacity. However, we cannot rule out some contribution of general antioxidant effect to the observed reduction in oxidative stress markers.

This study has several limitations that are inherent to fixed-sequence dose escalation studies. The dosing design does not allow us to fully attribute the observed effect on markers of oxidative stress to escalated dose or duration of CoQ_10_ supplementation and were unable to control for the effects of disease progression during the study. This study was conducted in patients undergoing thrice weekly hemodialysis, and did not include patients receiving alternate methods of dialysis, including peritoneal dialysis or daily dialysis. Since the exposures to uremic toxins in patients receiving other types of dialysis or dialytic regimens differ from our study population, we are unable to generalize our findings to these other dialysis regimens. The study examined relatively short-term effects of CoQ_10_ administration. Longer term CoQ_10_ supplementation could have some safety concerns, as CoQ_10_ could precipitate drug-drug interactions—in animal studies, CoQ_10_ supplementation has been shown to affect metabolism of theophylline [26], and *in vitro*, affect transport by P-glycoprotein, an intestinal drug transporter [27]. CoQ_10_ supplementation could also alter the oral bioavailability of digoxin and verapamil, both P-glycoprotein substrates.

In summary, CoQ_10_ appears to be safe and well tolerated in subjects receiving MHD and may reduce oxidative stress by improving mitochondrial function. Further studies are needed to investigate the potential metabolic and clinical benefits associated with longer term CoQ_10_ supplementation.

## Conclusions

Coenzyme Q_10_ supplementation improves mitochondrial coupling of respiration to oxidative phosphorylation, and decreases superoxide production in endothelial cells. CoQ_10_ may be especially beneficial in patients undergoing chronic hemodialysis, a population subject to increased oxidative stress. This is the first in vivo study in any patient population to demonstrate that plasma concentrations of isofurans are modifiable by any therapeutic antioxidant strategy and suggests that CoQ_10_ may improve mitochondrial function and decrease oxidative stress in patients receiving hemodialysis.

## Abbreviations

AIDS: Acquired immune deficiency syndrome
apoA-1: Apolipoprotein A-1
ATP: Adenosine Triphosphate
CoQ_10_: Coenzyme Q10
CoQ_9_: Coenzyme Q9
CPK: Creatine phosphokinase
ESRD: End stage renal disease
HD: Hemodialysis
HDL: High density lipoprotein
IL-6: Interleukin-6
INR: International normalized ratio
Met(O): Oxidized methionine
MHD: Maintenance hemodialysis
ROS: Reactive oxygen species
